# Combining Real-Time fMRI Neurofeedback Training of the DLPFC with *N*-Back Practice Results in Neuroplastic Effects Confined to the Neurofeedback Target Region

**DOI:** 10.3389/fnbeh.2016.00138

**Published:** 2016-06-28

**Authors:** Matthew S. Sherwood, Michael P. Weisend, Jessica H. Kane, Jason G. Parker

**Affiliations:** ^1^Wright State Research Institute, Wright State UniversityDayton, OH, USA; ^2^Department of Biomedical, Industrial, and Human Factors Engineering, Wright State UniversityDayton, OH, USA; ^3^Rio Grande NeurosciencesDayton, OH, USA; ^4^Department of Neuroscience, Cell Biology, and Physiology, Wright State UniversityDayton, OH, USA; ^5^Department of Neurology, Boonshoft School of Medicine, Wright State UniversityDayton, OH, USA

**Keywords:** fMRI, neurofeedback, DLPFC, *n*-back, plasticity, neuromodulation

## Abstract

In traditional fMRI, individuals respond to exogenous stimuli and are naïve to the effects of the stimuli on their neural activity patterns. Changes arising in the fMRI signal are analyzed *post-hoc* to elucidate the spatial and temporal activation of brain regions associated with the tasks performed. The advent of real-time fMRI has enabled a new method to systematically alter brain activity across space and time using neurofeedback training (NFT), providing a new tool to study internally-driven processes such as neuroplasticity. In this work, we combined *n*-back practice with fMRI-NFT of the left dorsolateral prefrontal cortex (DLPFC) to better understand the relationship between open- and closed-loop neuromodulation. FMRI data were acquired during both traditional *n*-back and NFT across five imaging sessions. Region-of-interest (ROI) and voxel-wise 2 × 2 within subjects ANOVAs were carried out to determine the effects of, and interaction between, training session and neuromodulation type. A main effect of training session was identified for only a single, highly focused cluster that shared spatial properties with the fMRI-NFT target region (left DLPFC). This finding indicates that combined open- and closed-loop neuroplastic enhancement techniques result in focal changes that are confined to the target area of NFT, and do not affect up- or down-stream network components that are normally engaged during working memory. Additionally, we identified a main effect of neuromodulation type for 15 clusters with significantly different activation between open- and closed-loop neuromodulation during training, 12 of which demonstrated higher activity during the open-loop neuromodulation. Our results, taken together with previous reports, indicate that fMRI-NFT combined with *n*-back practice leads to a highly focal volume exhibiting neuroplasticity without additional network effects.

## Introduction

Evidence shows that neuroplasticity follows specific learning-dependent changes in behavior (Kleim et al., 2004). The modulation of neuroplasticity has become a growing area of neuroscience research that holds potential to supplement current therapies for neurological disease (Jenkins and Merzenich, 1987; Wieloch and Nikolich, 2006) and enhance performance in healthy individuals (Garlick, 2002; Buschkuehl et al., 2008; Jaeggi et al., 2011; Jaušovec and Jaušovec, 2012). Neuroplasticity can be induced using electrical (McKinley et al., 2013; McIntire et al., 2014) or magnetic stimulation (Pascual-Leone et al., 1998; Fraser et al., 2002; Ziemann et al., 2002), but localization of the stimulation to specific brain regions can be challenging if the stimulation is delivered non-invasively (i.e., transcranially; Wassermann and Lisanby, 2001). Recent advances in functional magnetic resonance imaging (fMRI) acquisition and reconstruction times have enabled the use of real-time blood oxygen level-dependent (BOLD) signals combined with neurofeedback training (NFT) to induce and control localized neuroplasticity (Ogawa et al., 1990; Logothetis et al., 2001; Megumi et al., 2015).

In traditional fMRI, individuals respond to exogenous stimuli that activate specific brain regions or networks. During these tasks, the participants are naïve to the timing and location of the induced neural activity (open-loop neuromodulation). The external tasks are normally selected a priori to target specific functions and/or regions within the brain. Changes in the BOLD signal caused by the stimuli are studied to elucidate neural processes associated with each task. In real-time fMRI combined with NFT (fMRI-NFT), these same signals are captured immediately following each volume acquisition and reconstruction, and presented using visual or audio stimuli so that the subject may learn to modulate the signals at will (closed-loop neuromodulation). The application of fMRI-NFT to neuroplasticity is an active and growing field of research that can produce highly focal, non-invasive, and lasting changes in brain activity with no known side effects (Weiskopf et al., 2003; Birbaumer and Cohen, 2007; Daly and Wolpaw, 2008; Ros et al., 2010). Additionally, the ability to volitionally control neural activity, as trained in fMRI-NFT, holds the potential to be translated into in-home care routines, where they could be implemented without specialized equipment or professional supervision (Vaughan et al., 2006; Mak and Wolpaw, 2009).

The first behavioral modifications from fMRI-NFT were demonstrated by deCharms et al. (2005). They found a significant correlation between closed-loop neuromodulation of the rostral anterior cingulate cortex (supported by fMRI-NFT) and pain intensity ratings in an experimental group of healthy participants. The behavioral changes of decreased pain intensity and unpleasantness were significantly greater than any of the four control groups of healthy individuals. In addition, an experimental group of people diagnosed with chronic pain reported a significant decrease in average baseline pain levels compared to a diagnosis-matched control group. Since this study, fMRI-NFT has been applied to a broad range of clinical disorders. Ruiz et al. (2013) revealed participants diagnosed with schizophrenia were capable of learning volitional control of the BOLD signal. Additionally, they reported fMRI-NFT of the bilateral anterior insular cortex increased network connections among the insula cortex, amygdala, and medial prefrontal cortex, potentially facilitating the repair of abnormal neural networks in psychiatric populations. Cordes et al. (2015) present further evidence that schizophrenic patients engage different mental strategies and neural networks to perform closed-loop neuromodulation than healthy controls. Interestingly, differences in volitional control were found to not be caused by varying strategies. Other studies included patients diagnosed with Parkinson's disease (Subramanian et al., 2011), major depression (Linden et al., 2012; Young et al., 2014), and chronic tinnitus (Haller et al., 2010). These studies represent a fraction of the therapeutic potential of fMRI-NFT across a wide range of clinical disorders and its ability to target precise areas of the brain.

In previous work, we demonstrated the ability of healthy human subjects to gain increasing control over neural activity of the left dorsolateral prefrontal cortex (DLPFC), and showed that neuroplastic effects associated with increased control led to improved cognitive performance (Sherwood et al., 2016). In this current paper, we further evaluate data from this study to determine differences in plasticity induction between open- and closed-loop neuromodulation. BOLD data were acquired during both *n*-back practice and fMRI-NFT across 5 experimental sessions over 2 weeks. The target region for neurofeedback was individually selected during each session from an activation map produced from the *n*-back task. Due to the identification of the NFT feedback region from the *n*-back map, we hypothesized that similar changes in activity would occur across training in the neurofeedback target region between *n*-back practice and neurofeedback training. Additionally, in other work Buschkuehl et al. (2014) found significant increases in perfusion magnitude following 7 days of *n*-back practice in three distinct regions located in the frontal and occipital cortices. Increased network activity has also been reported in fMRI-NFT studies where open-loop neuromodulation was not performed (Caria et al., 2007; McCaig et al., 2011). Therefore, we further hypothesized a network of brain regions would be commonly activated during fMRI-NFT and *n*-back tasks, and when averaged, these regions would change similarly across sessions.

## Methods

### Participants

Eighteen (18) right-handed, healthy volunteers (10 males), ages 19–35 (mean 23.3) with normal or corrected-to-normal vision participated in the experiment. None of the participants were medicated for neurological or psychiatric disorders. Written informed consent was obtained prior to participation. This study was approved by the Institutional Review Board of Wright State University and the Air Force Surgeon General. Each participant was compensated equally for his or her involvement.

### Experimental design

**Figure 1:**
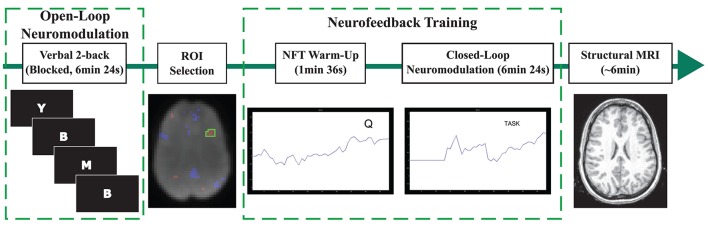
**Depiction of the procedures conducted during each session**. The *n*-back task was completed first. A session-specific, individualized region was selected from an activation map produced using data acquired during the *n*-back task. The warm-up NFT run involved viewing brain activity in real-time while performing the *n*-back task. Closed-loop neuromodulation entailed executing imagined tasks to modulate brain activity in the target region. Every session ended with the acquisition of a structural MRI.

The data presented here were collected during a previous study (Sherwood et al., 2016), and we refer the reader to the Methods Section of the previous paper for a detailed description of the experimental design. In brief, the experiment consisted of *n*-back practice and fMRI-NFT, which were both completed inside of the MR scanner (Siemens MAGNETOM Avanto 1.5 Tesla, Siemens, Erlangen, Germany). All subjects completed five sessions over a period of 2 weeks. Each session consisted of *n*-back practice followed by fMRI-NFT (Figure 1). NFT consisted of 2 runs: a brief 1 min-36 s warm-up session followed by a 6 min-24 s experimental session. BOLD data were acquired during the *n*-back and NFT protocols using a gradient-recalled-echo pulse sequence with a 64 × 64 element matrix, 24 slices parallel to the AC-PC plane, 4 × 4 × 5 mm^3^ voxel size, 1 mm slice gap, TR/TE = 2000/40 ms and a flip angle of 90°. Following training, a high-resolution T1-weighted structural image was acquired using a 3D Magnetization-Prepared Rapid Acquisition-Gradient-Echo (MPRAGE) sequence with a 256 × 256 element matrix, 160 slices oriented in the same plane as in the functional scans, 1 × 1 × 1 mm^3^ voxel size, TR/TE = 500/15 ms, and a flip angle of 15°.

#### *N*-back practice

The *n*-back task was conducted using a boxcar design with 48 s control and *n*-back blocks repeated four times. The *n*-back condition executed a 2-back variant using single letters from the English alphabet presented visually. Each letter appeared for 500 ms with an inter-stimulus interval of 2500 ms. A response was required for every stimulus to signify whether or not the conditions of the 2-back was satisfied; i.e., whether or not the current letter was the same as second previous in the list. The probability of the 2-back condition being satisfied on any trial was 40 percent. Response speed was encouraged by instructing participants to respond as quickly and accurately as possible. The control condition replaced the letters in the *n*-back task with a fixation point. During the control condition, participants were instructed to alternate between right and left responses with each presentation of a fixation point.

#### Region of interest selection

At each session, an activation map was produced from the BOLD data collected during the *n*-back task. A region-of-interest (ROI) in the left DLPFC was determined from the pattern of activity in the map localized using the superior surface of the ventricles. This ROI was used to derive the feedback signal for the subsequent neurofeedback. This procedure is described thoroughly in Sherwood et al. (2016).

#### Neurofeedback training

Neurofeedback training consisted of presenting a feedback signal from the specified ROI to the participant in real-time. The feedback signal was derived by pre-processing measured BOLD data from this ROI. Custom C++ and MATLAB (Mathworks, Inc., Natick, MA, USA) software implemented spatial filtering (5-point Gaussian low-pass kernel, full-width half-maximum of 9 mm) and motion correction (corrected to the first volume from the *n*-back task using a rigid-body 3-parameter model). The percent BOLD signal change was computed using the equation *(BOLD*_*current*_ − *BOLD*_*baseline*_*)/BOLD*_*baseline*_. The term *BOLD*_*current*_ represents the average signal in the ROI from the current time point and *BOLD*_*baseline*_ represents the average signal in the ROI from the first eight volumes of each run. The current feedback signal was determined by temporally-filtering (5-point Gaussian low-pass kernel consisting of only past components, sigma of 3 s) the percent BOLD signal change with the feedback signals from previous volumes. This feedback signal was presented to the participants using a continuously updated line plot scrolling from right-to-left.

During the warm-up NFT run, the participants performed the *n*-back task while viewing the feedback signal. A single repetition of the control and *n*-back blocks identical to the *n*-back practice was presented in addition to the plotted feedback signal. The control and *n*-back stimuli were presented on the upper-right side of the line plot. This run familiarized the participants with the feedback signal, the expected results of activating the voxels selected from the functional localizer, and the hemodynamic delay associated with this activation.

During the NFT run, participants performed four repetitions of 48 s rest and task blocks in a boxcar design. Every participant was instructed to lower the feedback signal during rest blocks and increase the feedback signal during task blocks. The feedback signal was plotted and task instructions were displayed on the upper right side of the plot. Suggestions were supplied to aid in up-regulating the BOLD signal in the left DLPFC prior to entering the MR scanner. These suggestions consisted of recalling their drive to the MRI site, the walk to the MRI room from the parking lot or a recent phone call, or performing mental math such as square root calculations. Also, the participants were informed not to use the response devices and to remain as still as possible.

### Data analysis

#### ROI-based analysis

A timeseries of the average BOLD signal of the left DLPFC ROIs during *n*-back practice and neurofeedback training was derived for the first and fifth session in the same method used to compute the feedback signal during NFT. A single explanatory variable (EV) was defined by convolving a boxcar model containing 48 s rest and task conditions with a pre-defined HRF. The model was fit to each ROI timeseries using a GLM by applying a weight of +1 to the EV, representative of activation of the target ROI. ROI t-statistics were computed by converting the resulting β-parameter using standard statistical transforms.

#### Whole brain analysis

BOLD data acquired during *n*-back practice and neurofeedback training from the first and fifth session was processed using the FMRIB Software Library (FSL; Smith et al., 2004; Woolrich et al., 2009). Individual (first-level) analyses were conducted on each of the 4D BOLD data sets. The data sets were pre-processed prior to the individual analyses. Both the pre-processing and first-level analysis were consistent with that described in Sherwood et al. (2016).

A group (higher level) analysis was carried out in FSL to perform a voxel-wise 2 × 2 (session by neuromodulation type) within subjects ANOVA. This analysis assumes the covariance between measures within-subject follows a compound symmetric structure (equal variance and intra-subject correlations being equal). This assumption is valid as the data were acquired in close proximity and fairly regularly sampled. On average, the first and fifth session were separated by ~225 ± 72 h. A mixed-effects modeling method capable of carrying the individual variances to the group analysis was implemented (Beckmann et al., 2003; Woolrich et al., 2004; Woolrich, 2008). The mixed-effects method allows inferences to be made about the populations from which our participants were selected but is less sensitive to activation than fixed-effects modeling. Three contrasts were created to identify voxels more active during session five than one (main effect of session), more active during neurofeedback training than *n*-back practice (main effect of neuromodulation type), and a larger change in activity from session one to five (5–1) for neurofeedback than *n*-back (interaction between session and neuromodulation type). Furthermore, parameter estimates from each of these contrasts underwent separate *F*-tests to explore the main effects of session and neuromodulation type, and their interaction. The resulting *Z*-statistic images for contrasts and main effects were thresholded using the clustering method outlined above.

## Results

### ROI-based analysis

**Figure 2:**
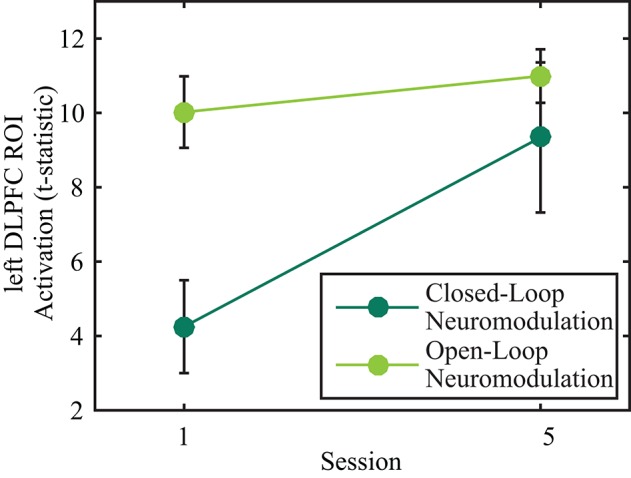
**Effects of open- (light green) and closed-loop (dark green) neuromodulation on activity in the left DLPFC ROI**. The main effects of session and neuromodulation type were significant (*p* < 0.05) while the interaction effect was not significant (*p* > 0.05). Error bars represent 1 SEM.

A 2 × 2 (session by neuromodulation type) within subjects ANOVA was performed on the left DLPFC ROI t-statistics using SPSS (IBM SPSS statistics for OSx version 21.0, IBM Corp., Amonk, NY). There were significant main effects of neuromodulation type [*F*_(1, 17)_ = 10.036, *p* = 0.006, Greenhouse-Geisser corrected degrees of freedom for the violation of sphericity] and session [*F*_(1, 17)_ = 6.605, *p* = 0.020, Greenhouse-Geisser corrected degrees of freedom for the violation of sphericity]. There was not a significant interaction effect [Figure 2; *F*_(1, 17)_ = 4.420, *p* = 0.051, Greenhouse-Geisser corrected degrees of freedom for the violation of sphericity]. *Post-hoc*, pairwise, Bonferroni-corrected comparisons indicated activity of the left DLPFC was significantly greater during open-loop neuromodulation (*p* < 0.001, two-tailed). However, no significant difference was found between open- and closed-loop neuromodulation at the fifth session (*p* > 0.05, two-tailed).

### Whole brain analysis

**Figure 3:**
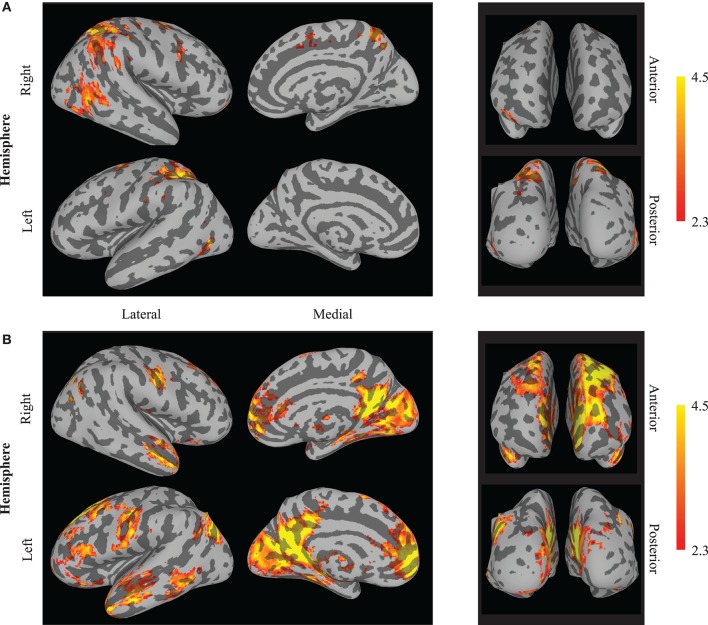
**Whole brain effects of neuromodulation type**. The results indicate increased activation (averaged between sessions one and five) during **(A)** open-loop neuromodulation (open-loop minus closed-loop neuromodulation) and **(B)** closed-loop neuromodulation (closed-loop minus open-loop neuromodulation). Images are displayed on an inflated brain surface. Only a subset of the differential activation appears from increased activity during *n*-back practice (open-loop neuromodulation) compared to closed-loop neuromodulation of the left DLPFC (see Table 1).

A 2 × 2 (session by neuromodulation type) within subjects ANOVA was performed using FSL. There was a significant (*Z* > 2.3) main effect of neuromodulation type appearing in 16 clusters, of which the center of gravity (COG) of 15 appear in the Talairach atlas (Table 1). It is important to note the cluster centered in the culmen of the vermis encompassed a large area. Local maxima of this cluster appeared bilaterally in the posterior cingulate of the limbic lobe and in the left cuneus of the occipital lobe. Due to the bi-directionality of the *F*-test, we cannot identify which clusters are more active during neurofeedback than *n*-back using this map alone. By assessing the contrast identifying voxels with significantly greater activation during open- than closed-loop neuromodulation, we found similar clusters centered in the left and right superior parietal lobule, the right middle temporal gyrus and middle frontal gyrus, and the left precentral gyrus (Figure 3A). This suggests these regions are more active during open-loop neuromodulation while the remaining are more active during closed-loop neuromodulation (Figure 3B).

**Table 1:** **Clusters identified with differential activation, on average, between ***n***-back practice and neurofeedback training**.

**Hemisphere**	**Lobe**	**Gyrus**	**Volume (mm^3^)**	**Cluster COG (mm)**		
				***X***	***Y***	***Z***
Right cerebellum	Anterior lobe	Culmen of vermis	133,480	4.29	−63	1.84
Left cerebrum	Frontal lobe	Medial frontal gyrus	85,104	−10.2	41.4	28.3
Right cerebrum	Parietal lobe	Superior parietal lobule	35,288	34	−52.8	50.6
Left cerebrum	Temporal lobe	Superior temporal gyrus	19,616	−50.9	−0.661	−5.69
Left cerebrum	Parietal lobe	Superior parietal lobule	19,072	−28.1	−52.6	50.2
Right cerebrum	Temporal lobe	Superior temporal gyrus	9720	49.9	8.88	−23.8
Left cerebrum	Temporal lobe	Middle temporal gyrus	9160	−40.4	−71.6	32.6
Right brainstem	Medulla		7328	5.95	−50.7	−46
Right cerebrum	Frontal lobe	Middle frontal gyrus	6504	26.5	−6.22	51.5
Left cerebrum	Temporal lobe	Middle temporal gyrus	4456	−60.6	−46	−8.5
Right cerebrum	Temporal lobe	Middle temporal gyrus	4392	44.9	−69.5	31.9
Right cerebrum	Frontal lobe	Precentral gyrus	4008	54.7	−7.2	29.9
Right cerebrum	Temporal lobe	Middle temporal gyrus	3960	58.5	−61.3	−0.348
Left cerebrum	Frontal lobe	Precentral gyrus	2904	−25.8	−7.25	53.3
Left cerebellum	Posterior lobe	Pyramis	2640	−29.7	−79.6	−35.1

*Only clusters with a COG appearing in the Talairach atlas are supplied. The highlighted clusters were found to be more active during open-loop neuromodulation of the left DLPFC. COG is given in MNI coordinates*.

**Figure 4:**
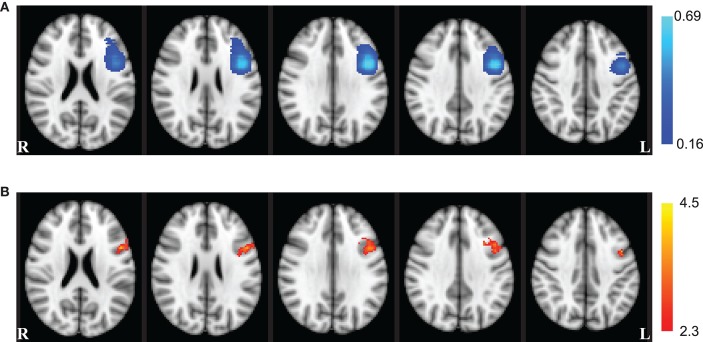
**Whole brain effects of session**. **(A)** The probability of voxel inclusion during NFT. Light blue voxels were included most frequently in the fMRI-NFT target region from the functional localizer, dark blue were included less frequently, and clear voxels were not included. **(B)** Whole brain ANOVA results for the main effect of session (red–yellow). The session effect showed a large overlap with the left DLPFC ROIs targeted for neurofeedback training. Axial slices are displayed in radiologic convention at the coordinates *z* = 22, 26, 30, 34, and 38 mm (left to right).

There was a significant (*Z* > 2.3) main effect of session in a single cluster (375 mm^3^) located in the left hemisphere centered on the MNI coordinate *x* = −45 mm, *y* = 9.97 mm, and *z* = 30.2 mm (Figure 4). This cluster overlaps parts of the inferior frontal gyrus, middle frontal gyrus, and precentral gyrus from the Talairach atlas (Talairach and Tournoux, 1988). A large portion of this cluster also overlapped with voxels appearing in left DLPFC ROIs utilized in fMRI-NFT. No significant interaction between neuromodulation type and session (*Z* < 2.3) was observed throughout the entire brain.

## Discussion

The exact mechanisms of behavior augmentations generated by fMRI-NFT are unknown and are currently being investigated (Birbaumer et al., 2013). One postulation is that behavior may be impacted by continuous fluctuations in brain state (Gilden et al., 1995; Boly et al., 2007). Yoo et al. (2012) used real-time fMRI to demonstrate the ability to recall scenes varies with the BOLD signal in the parahippocampal place area immediately prior to the presentation of a stimulus. They concluded an enhanced ability to remember scenes can be produced when, immediately prior to the onset of stimuli, the BOLD signal is in a specific state compared with a resting average. This result suggests neuromodulation based on exogenous tasks may have specific brain states that produce enhanced behavioral performance. From this, we theorize fMRI-NFT may alter behavior by enabling individuals to volitionally produce optimal brain states prior to executing tasks. Furthermore, volitional control trained from fMRI-NFT could enable these states to be induced without the use of external devices like transcranial direct current stimulation or transcranial magnetic stimulation.

Another postulation on the mechanisms of behavior modification is that closed-loop neuromodulation may enable individuals to more readily recruit task appropriate brain networks when processing stimuli. deCharms et al. (2005) found the same individuals who induced a greater change in rostral ACC activity through closed-loop neuromodulation also demonstrated a larger difference in pain intensity ratings. Other researchers found similar correlations between activity in the region targeted by fMRI-NFT and resultant behavioral changes (Linden et al., 2012; Sitaram et al., 2012; Ruiz et al., 2013). This correlation may be driven by individual differences in the recruitment of neural resources for task execution, not a generalized ability to prepare brain networks for specific tasks.

We sought to investigate the neural differences between open- and closed-loop neuromodulation. Elucidating these differences may help to understand how behavioral alterations are produced from closed-loop neuromodulation training. Previous results from this study indicated individuals were successful at learning volitional control of the left DLPFC from fMRI-NFT. This increased control led to performance improvements on untrained tasks beyond that of a control group (Sherwood et al., 2016). The results presented in this work indicate closed-loop neuromodulation produces higher activity in a far less focal network than open-loop neuromodulation. Ten of Fifteen clusters were found to have significantly higher activity during closed-loop neuromodulation compared to open-loop neuromodulation. Interestingly, although several areas demonstrated differential activity between open- and closed-loop neuromodulation, the change in activity from session one to five did not significantly differ between tasks in either the whole brain or ROI-based analyses.

The lack of an interaction effect in the ROI-based analysis confirms our hypothesis that similar changes in the left DLPFC would occur in both open- and closed-loop neuromodulation across training. This result was not surprising as the voxels selected for neurofeedback were determined from an activation map produced from the *n*-back task. The small area selected for neurofeedback may have impeded the participants' ability to volitionally control the left DLPFC. Future studies may assess target activity across open- and closed-loop neuromodulation when the neurofeedback ROI selection is not constrained by the activation pattern of the open-loop neuromodulation method, however, our study design does not allow us to examine this.

Although the ROI-based analysis did not indicate a significant interaction between neuromodulation type and training, *post-hoc* testing revealed that closed-loop neuromodulation had significantly lower left DLPFC activity at session one. By session five, there was not a significant difference in the activity of the left DLPFC between open- and closed-loop neuromodulation. This suggests that neurofeedback training brings left DLPFC activity to the level initially recruited by the *n*-back task, signifying *n*-back stimuli provide a better behavioral context to recruit the DLPFC while in the case of neurofeedback the participant must learn to reproduce that defined brain state. Previous published work from the dataset presented here indicated a linear increase in left DLPFC activity during closed-loop neuromodulation (Sherwood et al., 2016). However, our study was not focused on learning the upper limit of the volitional control over BOLD signals, therefore, future studies are necessary to determine the extent to which left DLPFC activity during closed-loop neuromodulation could surpass open-loop methods and if this would further enhance the observed behavioral effects.

Expanding upon our first hypothesis, the lack of an interaction effect in the whole brain analysis confirmed our second hypothesis that a common network of brain regions to open- and closed-loop neuromodulation would change similarly through training. Again, as the target region for neurofeedback was selected from the *n*-back activation map, this result was not unexpected. The *n*-back task drives a distributed pattern of activity, recruiting several regions (including the DLPFC) to support task performance (Owen et al., 2005). Although closed-loop neuromodulation may require the recruitment of additional regions to perform the task, the amount to which this distributed activity is common across individuals and sessions is unknown as these are most likely dependent upon the mental rehearsal technique implored by the participant to increase left DLPFC activity. Connectivity or network-based training, such as that presented by Koush et al. (2013), may be used to provide training for a functionally relevant pattern of activity which may be a better comparison to the *n*-back task.

Neurofeedback has been proposed to have the potential to not only alter brain activity but also induce neuroplastic processes (Birbaumer and Cohen, 2007; Daly and Wolpaw, 2008; Ros et al., 2010). Increased activity in a network of brain regions has been found separately with only *n*-back practice (Buschkuehl et al., 2014) and fMRI-NFT training that did not include open-loop neuromodulation (Caria et al., 2007; McCaig et al., 2011). However, the results of our study suggest a highly localized alteration in brain activity occurred when the *n*-back practice is combined with fMRI-NFT of the left DLPFC. Averaging across *n*-back and neurofeedback sessions, a single cluster exhibited a significant increase in activity between the first and fifth training session. This cluster was constrained to the area targeted for closed-loop neuromodulation. Similarly, the ROI-based analysis revealed a significant increase in activity across the training regimen. This finding was unexpected, as we anticipated similar changes to occur through overlapping portions of the networks utilized during both open- and closed-loop neuromodulation. Unfortunately, we are unable to determine if the differential activity within this cluster is due to *n*-back practice, fMRI-NFT, or their combination, although previous research suggests the result is not due to *n*-back practice or fMRI-NFT alone (Caria et al., 2007; McCaig et al., 2011; Buschkuehl et al., 2014). Future experiments will use paradigms where the effects of open- and closed-loop neuromodulation can be dissociated.

Our results provide further support for the use of fMRI-based closed-loop neuromodulation in combination with open-loop neuromodulation for the treatment of neurological disorders or enhancement of human performance. Neurological disorders may cause aberrant functioning of a specific brain area. Through neurofeedback training coupled with appropriate open-loop neuromodulation, focal plastic changes could be induced to return normal functioning to this area. In the case of human performance, areas playing critical roles in task execution may be targeted for neurofeedback training and task practice. Resulting focal increases in synaptic efficiency may improve the functioning of these regions, possibly aiding task execution and enhancing task performance. Future work in these domains should be conducted to address these claims.

## Author contributions

JP contributed to the design and provided the conception of and overall guidance to the project. MS, MW, JK, and JP contributed to the data collection, analysis, and interpretation of the data. MS, MW, and JS contributed to the initial drafting of the manuscript. MS produced the final artwork. MS and JP contributed to the development of the fMRI-NFT software. All authors contributed to the writing, revising, and approving of the manuscript, and are equally accountable for all aspects of the work.

## Funding

This work was supported by the Air Force Research Laboratory (AFRL) under the Neuroscience and Medical Imaging Program (contract FA8650-11-C-6157). Public release was approved with unlimited distribution (Distribution A; 88ABW-2014-5928). The opinions expressed herein belong solely to the authors. They do not represent and should not be interpreted as being those of or endorsed by the Department of Defense, or any other branch of the federal government.

### Conflict of interest statement

The authors declare that the research was conducted in the absence of any commercial or financial relationships that could be construed as a potential conflict of interest. The reviewer TR and handling Editor declared their shared affiliation, and the handling Editor states that the process nevertheless met the standards of a fair and objective review.
